# nf-core/marsseq: systematic preprocessing pipeline for MARS-seq experiments

**DOI:** 10.1093/bioadv/vbaf089

**Published:** 2025-05-23

**Authors:** Martin Proks, Jose Alejandro Romero Herrera, Jakub Sedzinski, Joshua M Brickman

**Affiliations:** Novo Nordisk Foundation Center for Stem Cell Medicine (reNEW), Department of Biomedical Sciences, University of Copenhagen, Copenhagen, 2200, Denmark; Center for Health Data Science, University of Copenhagen, Copenhagen, 2200, Denmark; Novo Nordisk Foundation Center for Stem Cell Medicine (reNEW), Department of Biomedical Sciences, University of Copenhagen, Copenhagen, 2200, Denmark; Novo Nordisk Foundation Center for Stem Cell Medicine (reNEW), Department of Biomedical Sciences, University of Copenhagen, Copenhagen, 2200, Denmark

## Abstract

**Motivation:**

Single sequencing technology (scRNA-seq) enables the study of gene regulation at a single cell level. Although many sc-RNA-seq protocols have been established, they have varied in technical complexity, sequencing depth and multimodal capabilities leading to shared limitations in data interpretation due to a lack of standardized preprocessing and consistent data reproducibility. While plate based techniques such as Massively Parallel RNA Single cell Sequencing (MARS-seq2.0) provide reference data on the cells that will be sequenced, the data format limits the possible analysis. Here, we focus on the standardization of MARS-seq analysis and its applicability to RNA velocity.

**Results:**

We have taken the original MARS-seq2.0 pipeline and revised it to enable implementation using the nf-core framework. By doing so, we have simplified pipeline execution, enabling a streamlined application with increased transparency and scalability. We have incorporated additional checkpoints to verify experimental metadata and improved the pipeline by implementing a custom workflow for RNA velocity estimation. The pipeline is part of the nf-core bioinformatics community and is freely available at https://github.com/nfcore/marsseq with data analysis at https://github.com/brickmanlab/proks-et-al-2023.

**Availability and implementation:**

We introduce an updated preprocessing pipeline for MARS-seq experiments following state-of-the-art guidelines for scientific software development with the added ability to infer RNA velocity.

## 1 Introduction

Over the past decade, a range of new single-cell RNA sequencing (scRNA-seq) protocols have been developed, each focusing on transcriptomic data collected using different approaches, each with unique advantages relevant to distinct experimental questions. Popular approaches include cell isolation using microfluidic devices, plate-based techniques, and nanopore-based strategies (Mereu *et al.* 2020). Each technique requires an additional pipeline that: (i) preprocesses raw reads, (ii) aligns them to a reference genome, (iii) performs demultiplexing to match an individual transcript to specific cells, and (iv) reports on the number of expressed genes per individual single cell in a tabular format known as count matrix. Microfluidic methods which use unique molecular identifiers (UMI) tag systems have become increasingly popular. These are usually processed by open-source tools, such as dropEst (for inDrop, Drop-seq) (Petukhov *et al.* 2018), kallisto bustool (for UMI generic) (Melsted *et al.* 2021), or umis (for UMI generic) (Svensson *et al.* 2017). Commercial platforms like 10X Chromium offer an out-of-the-box tool called cellranger (Zheng *et al.* 2017). Plate-based methods such as full-length SMART-seq2/3 or MARS-seq can be processed using zUMIs (Parekh *et al.* 2018) or StarSolo (Kaminow *et al.* 2021). To fully automate the preprocessing of the sequencing read, these tools can be wrapped in a pipeline using workflow managers such as Snakemake (Mölder *et al.* 2021) or Nextflow (Di Tommaso *et al.* 2017), to take full advantage of high-performance computing (HPC) resources. In this paper, we focus on the plate-based MARS-seq protocol (Keren-Shaul *et al.* 2019), which incorporates fluorescence-activated cell sorting (FACS) to investigate rare populations of cells. This approach reduces the occurrence of cell doublets and neighboring cell contamination, while keeping experimental costs low. We use two datasets with different properties; a large dataset which spanned 33 700 cells across 153 embryos that produced new insights into mouse gastrulation and a smaller dataset that focused on the differentiation of the endoderm lineage, identifying a rare, but pivotal transition state in lineage specification. Unfortunately, setting up and executing the original pipeline for MARS-seq experiments using MARS-seq2.0 also proved to be a challenging task, as the pipeline required custom references genome for aligner, a specific folder structure with the raw files split to smaller ones, and missing scaling capabilities. Despite thorough documentation, the setup and execution of the pipeline was not trivial and could be simplified. To avoid these problems and make this technology more accessible, we streamlined the execution for noncomputational researchers and extended its portability, and execution by utilizing nf-core framework (Ewels *et al.* 2020).

## 2 Methods

**Figure 1.**
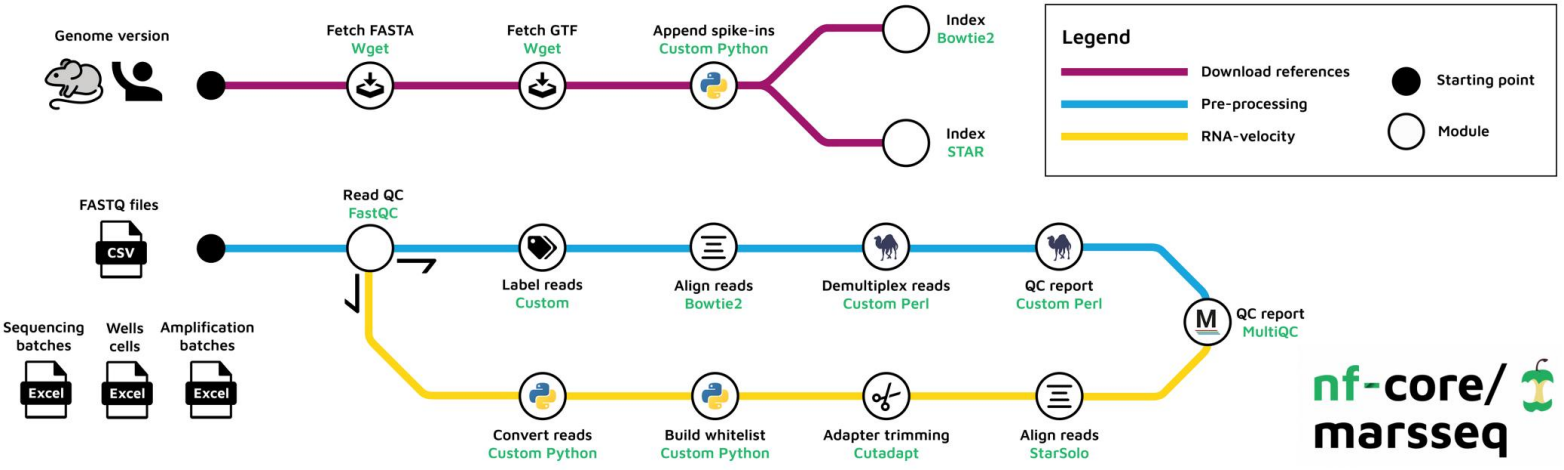
Overview of nf-core/marseq v1.0.3. The pipeline supports both mouse and human genomes. It consists of three workflows: (i) reference building, (ii) construction of count matrix, and (iii) RNA velocity estimation. At the end of each run, all quality control statistics are summarized with MultiQC.

The nf-core/marseq pipeline (v1.0.3) is straightforward to execute and involves two main steps. First, the building of the necessary reference indexes for a designated genome (in this case, either human or mouse). Second, the pipeline aligns the raw reads and generates a count matrix which is then utilized for further downstream analysis. An overview of all available workflows can be found in Fig. 1.

### 2.1 Reference building

The first step of the pipeline, run with the - -build_references flag, automates the incorporation of ERCC spike-ins into the reference of choice. The original MARS-seq2.0 pipeline provided only the custom mouse (mm9) reference for download. The custom reference is required due to the use of External RNA Controls Consortium (ERCC) spikes as controls for accurate gene expression measurements (Pine *et al.* 2016). The incorporation of these ERCC spike-ins requires conversion of ERCC sequences (*N* = 92) to FASTA format and manual concatenation to the reference genome (Tanaylab 2019). In our pipeline, we have automated this process using an argument which specifies the organism of interest, in order to download a reference genome (FASTA) and gene annotation (GTF) from the GENCODE database. The pipeline then appends the ERCC spike-in sequences and builds reference indexes for required aligners. Bowtie2 (Langmead and Salzberg 2012) is set as the default aligner, following the original MARS-seq2.0 pipeline. The workflow is summarized in the upper panel of Fig. 1. Reference building can be executed using a simple one-line command:


nextflow run nf−core/marsseq−r 1.0.3−profile test --build_references --genome mm10 --velocity

### 2.2 Pipeline execution

Running the nf-core/marsseq pipeline after generating custom references as described in the previous step, requires a design file which contains information about the specific experiment. Each MARS-seq experiment is comprised of paired end raw sequencing (FASTQ) reads with experimental annotations based on sequencing batches (SB) that are described in the seq_batch.txt file. Each SB contains multiple amplification batches (AP, amp_batch.txt), and the wells contained within that batch (WS), described in (wells_cells.txt). The original pipeline required these files in tabular separated format (tsv). However, based on our experience, it is much easier to create and update these files using Microsoft Excel (xlsx). Therefore, we have developed a set of custom Python scripts that convert Excel files into the required tabular format and validate the resulting files, checking for any mistakes. If a mistake is detected, the pipeline stops and notifies the user with an appropriate error message. These additional validation steps were not included in the original MARS-seq2.0 workflow and therefore produced inconsistent error messages without terminating pipeline execution. To maintain consistency with the previous pipeline, we have kept metadata changes to a minimum. Our new pipeline documentation includes a prefilled metadata template to facilitate its use. Assuming that all input files are correct, the pipeline then performs quality control on raw reads using FastQC (Andrews 2012) and assigns barcode labels to the sequence reads (label reads module). The FASTQ files are then divided into subsets of 4 000 000 reads per file to avoid Bowtie2’s memory restrictions during alignment. Poor-quality reads are discarded and contaminating adapter sequences are trimmed. In this workflow, the alignment is performed using the Bowtie2 aligner (align reads module). The aligned reads are subsequently demultiplexed based on labeled barcodes, followed by the generation of a count matrix (demultiplex module). Finally, quality control (QC) reports are generated and summarized using MultiQC (Ewels *et al.* 2016). We had to adjust the original MARS-seq2.0 preprocessing Perl scripts in order to convert them into individual nextflow modules. For instance, most of the scripts were internally re-reading configuration scripts which is done at the beginning in our pipeline and can be supplied immediately as an input parameter instead. Lastly, we simplified and fixed some of the quality control plotting functions that were not rendering figures. Unless specified otherwise, all outputs are stored in the results folder (Fig. 1, preprocessing workflow). The nf-core/marsseq pipeline can be executed as follows:


nextflow run nf−core/marsseq−r 1.0.3−profile test --input samplesheet.csv

Our pipeline was developed utilizing the nf-core/tools template (Ewels *et al.* 2020), a community-curated python package adhering to best-practice and using Nextflow as a underlying workflow manager. Taking advantage of Nextflow, the pipeline can be executed on local computer, High Performance Computing (HPC) and cloud providers (such as Amazon and Google). Tracking of the pipeline can be done online using the open-source external service Nextflow Tower (Di Tommaso *et al*. 2017). Container technologies such as Docker, Podman, or Singularity are also supported to ensure future reproducibility of the pipeline and pre-processed data. Lastly, nf-core/marsseq implements extra steps that enable the estimation of RNA velocity, which was not possible with pre-existing MARS-seq2.0 pipelines.

### 2.3 RNA velocity estimation

**Figure 2.**
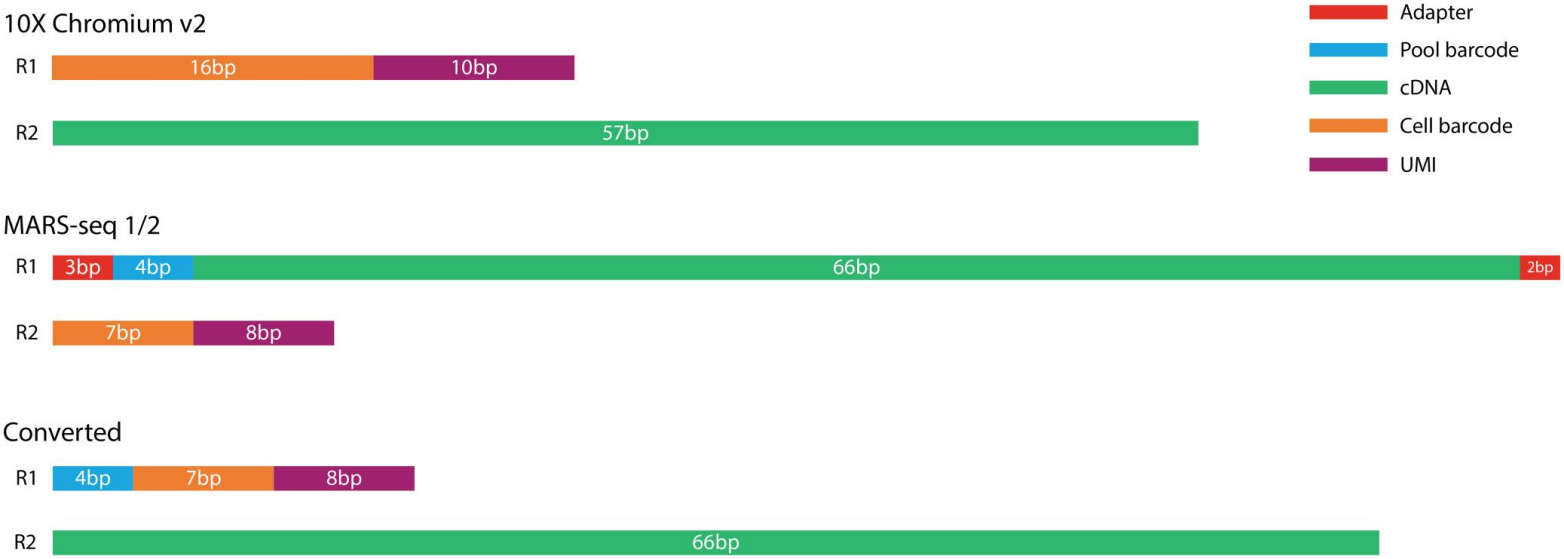
Raw reads structure. (10X) R1: CB (16 bp) + UMI (10 bp); R2: cDNA (57 bp). (**MARS-seq**) R1: LA (3 bp) + PB (4 bp) + cDNA (66 bp) + RA (2 bp); R2: CB (7 bp) + UMI (8 bp). (**Converted**) R1: PB (4 bp) + CB (7 bp) + UMI (8 bp); R2: cDNA (66 bp). Legend: CB (cell barcode), UMI (Unique Molecular Identifier), LA (left adapter), PB (pool barcode), RA (right barcode).

RNA velocity is a powerful method for predicting cellular dynamics and future cell states during differentiation. It does so by modeling the relationship between the observed number of spliced and unspliced transcripts (La Manno *et al.* 2018, Bergen *et al.* 2020). This process, however, requires a splice aware aligner such as kallisto, Alevin-fry (He *et al.* 2022), or StarSolo. Another option is to use already aligned bam files and process them with the Python package velocyto (La Manno *et al.* 2018). Neither of these options supports the unique MARS-seq double barcoding. To overcome this limitation, we convert MARS-seq library reads to a compatible 10X 3’ gene expression format. MARS-seq is a paired-end method where read 1 (R1) consists of a left adapter (LA, 3 bp), a pool barcode (PB, 4 bp), and cDNA (68 bp). Read 2 (R2) contains a cell barcode (CB, 7 bp) and a UMI (8 bp). All barcodes are stored in the header of each individual read. In comparison, for sequenced 3’ 10X libraries, R1 consists of a CB and a UMI, while the cDNA is stored in R2. To mimic the 10X format, we merged PB, CB and UMI sequences to generate a new R1 and moved the adapter free cDNA to R2 (Fig. 2).

Based on previous work benchmarking aligners for scRNA-seq (Du *et al.* 2020, Soneson *et al.* 2021), we decided to use StarSolo to estimate unspliced reads. To demultiplex the reads, the pipeline generates a file containing all valid cell barcodes containing concatenated PB with CB (11 bp) called whitelist.txt, which is required by StarSolo. We have also adjusted the default parameters to comply with the updated barcoding scheme as follows: - -soloType CB_UMI_Simple - -soloCBstart 1 - -soloCBlen 11 - -soloUMIstart 12 - -soloUMIlen 8 - -soloFeatures Gene GeneFull SJ Velocyto. To enable the estimation of spliced, unspliced and ambiguous transcripts, one simply needs to append the below velocity flag:


nextflow run nf−core/marsseq−r 1.0.3−profile test --input design.csv --velocity

## 3 Results

**Figure 3.**
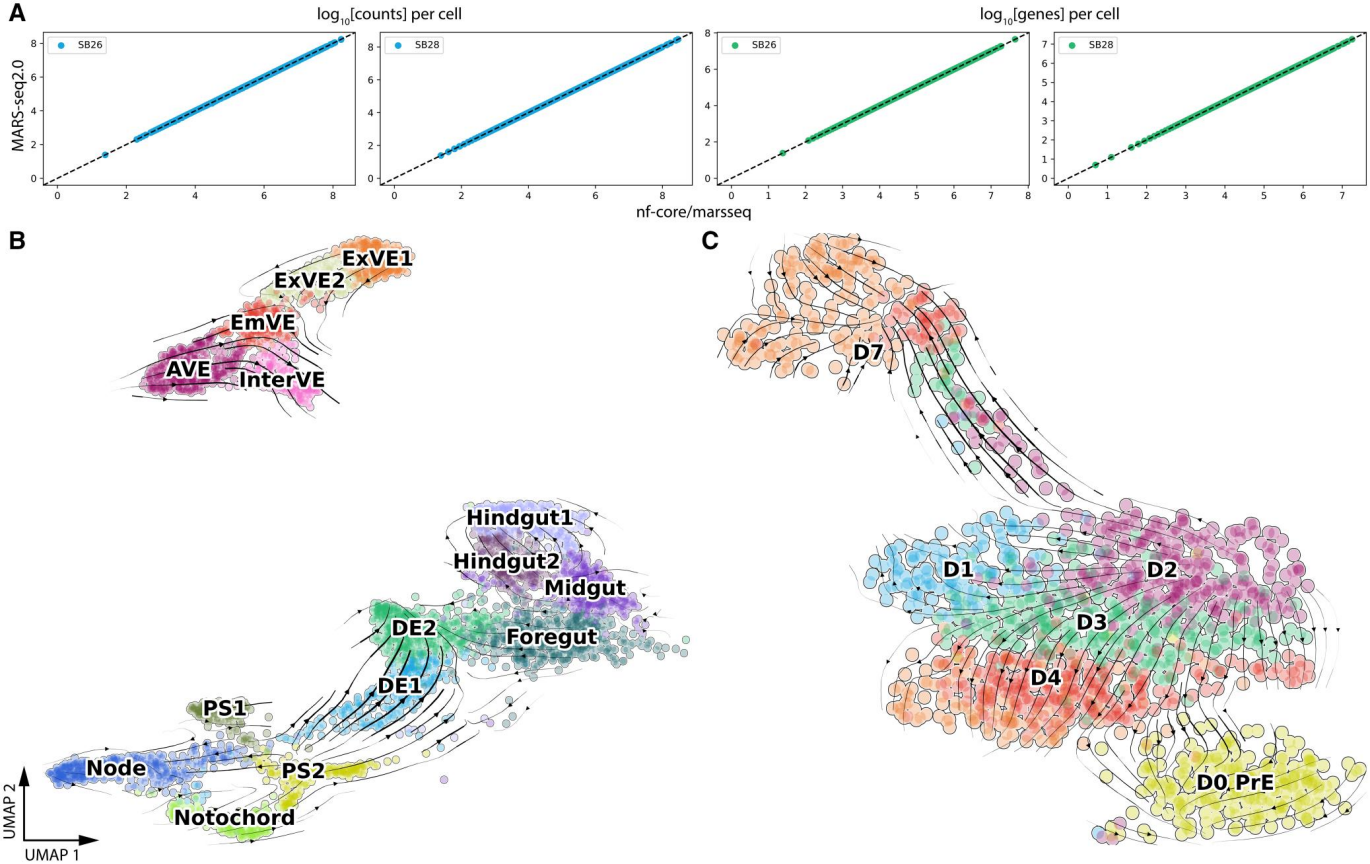
RNA velocity estimation. (A) Scatter plot comparing number of counts per cell (left blue panels) and number of genes detected per cell (right green panels) between the original MARSseq2.0 and nf-core/marseq pipelines. The dotted line represents a perfect fit (Pearson correlation of 1). (B) Visualization of RNA velocity computed from (Rothová *et al.* 2022). (C) Re-analysis and visualization of RNA velocity estimation of in vitro PrE differentiation from (Perera *et al.* 2022).

To ensure the new nf-core/marsseq (v1.0.3) pipeline could be used as a drop-in replacement for the MARS-seq2.0 pipeline, we quantified number of detected transcripts for two small test datasets (SB26 and SB28) provided in the MARS-seq2.0 documentation. For both pipelines, we used the newly built mm10 mouse reference genome for mapping. In the case of transcript quantification, we found a high concordance between the two pipelines, for both raw counts and number of genes per individual cell (*ρ *= 0.999) (Fig. 3A). Next, we validated RNA velocity inference, a function not performed by the MARS-seq2.0 analysis scripts, using two in-house generated datasets. The first published in Rothová *et al.* (2022) and the second involved the use of published data (Perera *et al.* 2022) to generate new inferences. In prior published work, we investigated in vivo endoderm differentiation using a FOXA2-Venus reporter to purify endoderm from mouse embryos. We had identified an intermediate cell population that provided a bridge for the conversion of extra-embryonic visceral endoderm, previously thought to only differentiate into visceral yolk sac, into embryonic endoderm. The embryonic endoderm comprises of progenitor populations for the gut associated organs. Using Partition-Based Graph Abstraction (Wolf *et al.* 2019) trajectory inference and RNA velocity, we were able to describe the route taken by visceral endoderm, across this bridge and onwards. To determine whether this route could be recapitulated in vitro, we differentiated naïve extra-embryonic endoderm stem cells (Anderson *et al.* 2017), in 3D cultures to produce gut spheres, which were characterized by scRNA-seq using MARS-seq. As with the in vivo data, we used trajectory inference, to determine the route of endoderm differentiation in vitro. At the time of writing Rothová *et al.* (2022), the nf-core/marsseq was still in development. We therefore published the RNA velocity workflow as a bash script which was later incorporated into our new pipeline as a module. In short, the original FASTQ files were re-aligned to mm9 using STARSolo v2.7.9a to infer spliced and unspliced reads. Count matrices were merged with the already analyzed dataset and further processed using scVelo (Bergen *et al.* 2020). We projected the estimated velocities onto the original UMAP visualization, as shown in Fig. 3B. In Perera *et al.* (2022) the link between lineage priming, cell cycle and lineage specification was explored by a combination of imaging and scRNA-seq. We found that during primitive endoderm (PrE) differentiation, the G1 phase of the cell cycle is increased, despite an overall enhanced rate of cell division or reduction in cell cycle length. This increase in G1 was observed both in vivo and in vitro for PrE differentiation. Phase of the cell cycle at different points in lineage specification were determined by a combination of live imaging and gene expression analysis, utililizing a double reporter embryonic stem cell (ESC) line to provide a florescent signature of cell fate choice. This reporter contained an epiblast/ESC specific Sox2-GFP and PrE specific Hhex-mCherry. Time lapse imaging of this reporter indicated a clear lineage bifurcation that correlated with changes in overall cell cycle length. To address whether we could detect changes in the cell cycle that correlated with differentiation in scRNA-seq, ESCs were differentiated in vitro for 7 days to PrE using the protocol described in Anderson *et al.* (2017) and sorted based on reporter expression. At each time point populations of cells expressing different levels of the two reporters were sequenced using MARS-seq. We preprocessed the data with the MARS-seq2.0 pipeline using mm9 genome as reference. We identified groups of cells that either differentiated toward the PrE fate or remained in an epiblast like state (NEDiff) fate. As the original dataset contained equal proportions of each sorted population regardless of its representation in the culture at that time point, the distribution of NEDiff and PrE cells sequenced did not reflect the increasing proportion of these cultures that were becoming PrE. The original analysis also did not address whether cells were progressing through PrE differentiation via a progressive alteration of cell cycle. To address whether PrE differentiation progressed from ESCs into PrE in via a clear trajectory that included the previously observed alterations to the cell cycle, we reprocessed the original FASTQ files with the nf-core/marsseq pipeline using the mm10 reference genome. The newly generated raw count matrices were merged and processed using scanpy (Wolf *et al.* 2018), while RNA velocity analysis was carried out using scVelo, projecting velocities onto a newly recomputed UMAP. Our new analysis supports time lapse imaging and marker analysis in Perera *et al.* (2022), demonstrating a clear bifurcation producing two trajectories (Fig. 3C).

## 4 Discussion and conclusion

In this paper, we have developed a robust and reproducible preprocessing Nextflow pipeline for MARS-seq experiments using nf-core tools. To achieve this, we took the original MARS-seq2.0 pipeline and broke it down into individual preprocessing steps, wrapping them into modules using Nextflow’s DSL2 syntax. Our pipeline consists of two main workflows: reference building and execution. We simplified the required input files as well as integrating a set of checkpoints for their validation, making the pipeline sufficiently simple to run for molecular biologists without extensive computational experience. By using Nextflow as workflow manager, the pipeline can be executed both on cloud as well as in-house HPC systems. Additionally, it supports standard containerized solutions that safeguard reproducibly. Lastly, execution can be tracked online by integrating it with Nextflow Tower. At the time of submission of this work we identified a tool that was designed to interconvert different sequencing formats (Battenberg *et al.* 2022); however, although this appears a robust tool for multiple applications, we were unable to use it to convert MARS-seq2.0 to 10X format. As a major limitation of MARS-seq was its incompatibility with RNA velocity measurements, we have developed a workflow for RNA velocity inference using MARS-seq data. This pipeline has been applied to several datasets in our lab, where it has successfully identified novel differentiation trajectories, both in vivo (Rothová *et al.* 2022) and in vitro (Perera *et al.* 2022) (Fig. 3). This is achieved by converting MARS-seq reads into 10X v2 format and customizing the parameters for the STARSolo aligner as a means to estimate unspliced read counts. This approach has produced important insight into novel routes of endoderm differentiation. Currently, our pipeline supports the original workflow using the Botwie2 aligner and can generate an additional count table using the RNA velocity workflow. However, based on our experience and similar findings from Du *et al.* (2020), we recommend using the STARSolo aligner for speed. However, the biggest bottleneck of the pipeline is the conversion to 10X format, which is limited by the I/O speed of hard drives.

## Data Availability

The in vitro files were downloaded from GSE200534 with data analysis available at https://github.com/brickmanlab/proks-et-al-2023 and processed data deposited on Zenodo [10.5281/zenodo.8016374]. The in vivo data were downloaded from Zenodo [10.5281/zenodo.6566016].
